# Delivery of incretin receptor agonists to the brain

**DOI:** 10.1002/alz.089829

**Published:** 2025-01-09

**Authors:** Elizabeth Rhea

**Affiliations:** ^1^ University of Washington, Seattle, WA, USA; VA Puget Sound Health Care System, Seattle, WA USA

## Abstract

**Background:**

Incretin receptor agonists (IRAs) are synthetic peptides that have beneficial effects within the brain. We investigated IRA brain uptake following two routes of delivery: across the blood‐brain barrier (BBB) and following intranasal (IN) delivery.

**Method:**

Radiolabeled IRAs were delivered intravenously or intranasally into CD‐1 mice and uptake by or distribution throughout the brain was assessed. Additionally, distribution following IN delivery was assessed in APP/PS1 and wild‐type male and female mice.

**Result:**

We identified many, but not all, IRAs are able to cross the BBB, and some have faster transport rates than others. We also identified IRAs can be delivered intranasally and that distribution does not majorly differ in a mouse model of Alzheimer’s disease (AD).

**Conclusion:**

The ability for IRAs to access the brain directly shows promise for AD therapeutics and will advance the design of future therapeutics. The IRAs that have a greater brain uptake should be taken into consideration for testing as AD as they would have the ability to act quickly and directly within the brain.

